# Alloantigen-activated (AAA) CD4^+^ T cells reinvigorate host endogenous T cell immunity to eliminate pre-established tumors in mice

**DOI:** 10.1186/s13046-021-02102-6

**Published:** 2021-10-08

**Authors:** Kazuhiro Mochizuki, Shogo Kobayashi, Nobuhisa Takahashi, Kotaro Sugimoto, Hideki Sano, Yoshihiro Ohara, Shin Mineishi, Yi Zhang, Atsushi Kikuta

**Affiliations:** 1 Department of Pediatric Oncology, Fukushima Medical University Hospital, 1 Hikarigaoka, 960-1295 Fukushima City, Japan; 2 Department of Basic Pathology, Fukushima Medical University, Fukushima, Japan; 3 Department of Medicine, Penn State Hershey Medical Center, Hershey, Pennsylvania USA; 4 Fels Institute for Cancer Research and Molecular Biology, Temple University, Philadelphia, USA; 5 Department of Cancer and Cellular Biology, Temple University, Philadelphia, USA

**Keywords:** Cancer immunotherapy, Cancer vaccine, Allogeneic CD4^+^ T-cell, Endogenous CD8^+^ cytotoxic T-cell, Dendritic cell

## Abstract

**Background:**

Cancer vaccines that induce endogenous antitumor immunity represent an ideal strategy to overcome intractable cancers. However, doing this against a pre-established cancer using autologous immune cells has proven to be challenging. “Allogeneic effects” refers to the induction of an endogenous immune response upon adoptive transfer of allogeneic lymphocytes without utilizing hematopoietic stem cell transplantation. While allogeneic lymphocytes have a potent ability to activate host immunity as a cell adjuvant, novel strategies that can activate endogenous antitumor activity in cancer patients remain an unmet need. In this study, we established a new method to destroy pre-developed tumors and confer potent antitumor immunity in mice using alloantigen-activated CD4^+^ (named AAA-CD4^+^) T cells.

**Methods:**

AAA-CD4^+^ T cells were generated from CD4^+^ T cells isolated from BALB/c mice in cultures with dendritic cells (DCs) induced from C57BL/6 (B6) mice. In this culture, allogeneic CD4^+^ T cells that recognize and react to B6 mouse-derived alloantigens are preferentially activated. These AAA-CD4^+^ T cells were directly injected into the pre-established melanoma in B6 mice to assess their ability to elicit antitumor immunity *in vivo*.

**Results:**

Upon intratumoral injection, these AAA-CD4^+^ T cells underwent a dramatic expansion in the tumor and secreted high levels of IFN-γ and IL-2. This was accompanied by markedly increased infiltration of host-derived CD8^+^ T cells, CD4^+^ T cells, natural killer (NK) cells, DCs, and type-1 like macrophages. Selective depletion of host CD8^+^ T cells, rather than NK cells, abrogated this therapeutic effect. Thus, intratumoral administration of AAA-CD4^+^ T cells results in a robust endogenous CD8^+^ T cell response that destroys pre-established melanoma. This locally induced antitumor immunity elicited systemic protection to eliminate tumors at distal sites, persisted over 6 months *in vivo*, and protected the animals from tumor re-challenge. Notably, the injected AAA-CD4^+^ T cells disappeared within 7 days and caused no adverse reactions.

**Conclusions:**

Our findings indicate that AAA-CD4^+^ T cells reinvigorate endogenous cytotoxic T cells to eradicate pre-established melanoma and induce long-term protective antitumor immunity. This approach can be immediately applied to patients with advanced melanoma and may have broad implications in the treatment of other types of solid tumors.

**Supplementary Information:**

The online version contains supplementary material available at 10.1186/s13046-021-02102-6.

## Background

Immunotherapies developed over the past decades have been promising approaches for the treatment of patients with advanced cancers [1–6]. However, the actual benefits of these immunotherapies have been achieved only in a limited number of cancer patients [7, 8]. To overcome intractable cancers, induction of strong endogenous antitumor immunity may represent the desired strategy. Cancer vaccines have been developed to induce endogenous immune responses [9, 10]. However, clinical studies of both peptide- and autologous dendritic cell (DC)-based vaccines have shown limited efficacy with respect to the induction of an objective clinical response rate and/or survival [1, 9–24]. These observations indicate that therapeutic vaccines using autologous immune cells seem to be insufficient for inducing potent endogenous immune responses against pre-established cancers.

Since the 1960 s, several groups of investigators have tried to induce host antitumor immunity by adoptive cell transfer of allogeneic lymphocytes without utilizing hematopoietic stem cell transplantation (HSCT), and they called the favorable response elicited by this an “allogenic effect” [25–27]. In these studies, allogeneic mononuclear cells or lymphocytes were transferred into allogeneic hosts and induced some clinical benefits [28–31]. More recently, specialized allogeneic lymphocytes, namely mitomycin C-inactivated allogeneic lymphocytes, or effector/memory CD4^+^ T helper-1 (Th1) lymphocytes that were non-specifically activated by CD3/CD28 antibodies have been used [32–37]. According to these studies, allogeneic CD4^+^ T cells administered *in vivo* have a unique potential to enhance host immune responses as a cell adjuvant. However, to confer antitumor specificity to the host, these strategies often require additional treatments such as the corresponding tumor cell lysate vaccinations and/or chemotherapy prior to the administration of allogeneic CD4^+^ T cells [33–37]. Although host antigens that elicit immune responses of transferred allogeneic CD4^+^ T cells can induce anti-tumor activity, only a small fraction of these CD4^+^ T cells are reactive against the host antigens. Thus, new approaches that can augment the generation of host-reactive allogeneic CD4^+^ T cells are important for inducing greater endogenous antitumor immunity in tumor-bearing hosts.

In the present study, we generated host-antigen-reactive CD4^+^ T cells from allogeneic CD4^+^ T cells in cultures activated using host-derived DCs. These alloantigen-activated CD4^+^ (AAA-CD4^+^) T cells were directly injected into the pre-established tumor to assess their ability to elicit antitumor immunity *in vivo*.

## Materials and methods

### Study design

This study was conducted using a mouse model of melanoma. AAA-CD4^+^ T cells were obtained from BALB/c mouse CD4^+^ T cells in cultures activated using host B6 mouse-derived DCs. Nine days after subcutaneous injection of B16F1 melanoma, AAA-CD4^+^ T cells were injected into the pre-established tumor in host B6 mice. There were two experimental endpoints. First, for the endpoint of tumor growth and survival experiments, sample sizes and the number of independent experiments was determined by the availability of *in vitro* activated cells and previous studies with similar types of experiments. The PBS-injected group served as the control. Second, for the endpoint of T cell immune response experiments, tumor-infiltrating immune cells were isolated at 4 and 24 h after intratumoral injection of AAA-CD4^+^ T cells. The host CD4^+^ T cells activated using syngeneic DCs (auto-CD4^+^ group) and PBS-injected group were used as controls. The animals were randomized into different groups after tumor cell inoculation. The group sizes and the number of independent experiments are indicated in the figures or figure legends. Blinded tests were not performed in this study. No outliers were excluded from this study.

### Mice, cell lines, antibodies, and flow cytometry analysis

B6 (H-2^b^, 8 to 10 weeks, female) and BALB/c (H-2^d^, 8 to 10 weeks, female) mice were purchased from CLEA Japan Inc. (Tokyo, Japan). 129 × 1/SvJJmsSlc (H-2^b^, 8–10 weeks, female) mice were purchased from Japan SLC (Shizuoka, Japan). CD4 knockout mice (B6.129S2-*Cd4*^*tm1Mak*^/J) were purchased from The Jackson Laboratory (Bar Harbor, ME, USA; Stock No: 002663). The B16F1 melanoma cell line (derived from B6 mice) was purchased from RIKEN BRC Cell Bank (Ibaraki, Japan; #RCB2649.00). All antibodies used for cell isolation and immunofluorescence staining were purchased from BioLegend (San Diego, CA, USA). Microbead-conjugated antibodies and streptavidin were purchased from Miltenyi Biotec (Auburn, CA, USA). Recombinant mouse granulocyte macrophage colony-stimulating factor (GM-CSF; #415-ML-010), stem cell factor (SCF; #455-MC), interleukin-4 (IL-4; #404-ML-010), and tumor necrosis factor-α (TNF-α; #410-MT-010) were purchased from R&D Systems (Minneapolis, MN, USA). Recombinant mouse FMS-like tyrosine kinase 3 ligands (Flt3L; #200-06-100 µg) and human IL-2 (#100-12-100 µg) were purchased from Shenandoah Biotechnology (Warwick, PA, USA). Flow cytometry (FCM) and flow cell sorting were performed using FACSCanto™ II (Becton Dickinson, Franklin Lakes, NJ, USA) and FACSAria™ II (Becton Dickinson), respectively.

### Generation of dendritic cells in vitro

The method for *the in vitro* generation of Flt3L-induced DCs (Flt3L-DCs) has been described previously [38, 39]. Briefly, mouse bone marrow (BM) mononuclear cells were cultured in RPMI-1640 medium containing 10 % fetal bovine serum (FBS), Flt3L (50 ng/mL), and SCF (5 ng/mL). Ten days later, the immature DCs were incubated with lipopolysaccharide (LPS, 100 ng/mL, Sigma-Aldrich, St. Louis, MO, USA; #L4391-1MG) and R848 (resiquimod, 100 ng/mL; InvivoGen, San Diego, CA, USA; #tlrl-r848), respectively, for 6 h to activate Toll-like receptor (TLR) 4 and TLR7/8 signaling. The cells were then harvested for further experiments. Generation of GM-CSF-induced murine DCs (GM-DCs) *in vitro* has been described previously [38, 39]. Briefly, c-kit-positive hematopoietic stem cells purified from the BM were cultured in RPMI-1640 medium containing 10 % FBS, GM-CSF (10 ng/mL), SCF (10 ng/mL), IL-4 (2.5 ng/mL), and TNF-α (4 ng/mL). On day 10 of culture, the DCs were stimulated with LPS (100 ng/mL) and R848 (100 ng/mL) for 6 h. Activated DCs were then harvested for experimental assays.

### Mixed lymphocyte reaction

CD4^+^ T cells and/or CD8^+^ T cells were isolated from the spleens of B6 and/or BALB/c mice using microbead-conjugated antibodies (MiniMACS™; Miltenyi Biotec; #130-049-201, and #130-049-401). The purity was consistently > 92 %. T cells were cultured in 96-well U-bottom plates (Corning, Corning, NY, USA; #3799), complete RPMI-1640 medium containing human IL-2 (200 IU/mL), and activated DCs. The ratio of T cells to DCs ranged from 2:1 to 4:1. T cells were cultured for three days at 37 °C with 5 % CO_2_, and then collected and injected into the experimental mice. The number of injected T cells was 2 × 10^6^/mouse.

### Tumor-bearing mouse models

B6 mice were subcutaneously injected with B16F1 cells suspended in 100 µL of PBS on day zero. The number of injected tumor cells was1.0 × 10^6^/ mouse, except if specified otherwise. Subsequently, *ex vivo* activated DCs or T cells were injected into B6 mice bearing pre-established B16F1 melanoma on the ninth day after tumor inoculation. Cell products were suspended in 50 µl of PBS and were then directly injected into the tumor using a 1 mL syringe with a 27 gauge needle. Several preclinical and clinical studies have suggested that local immunization is important for eliciting local immune responses [40–43]. In some experiments, host CD8^+^ T cells and natural killer (NK) cells were individually depleted using anti-mouse CD8a (BioXcel, New Haven, CT, USA; # BE0004-1) and anti-mouse NK1.1 (BioXcel; # BE0036). Tumor size, changes in physical conditions, and activity of the animals were monitored every two to three days from four or six days after the tumor inoculation until the experimental endpoints. Tumor dimensions were measured using calipers, and the tumor volume was calculated using the following formula: Tumor volume (mm^3^) = (length × width^2^)/2. All mice with a tumor diameter exceeding 18 mm were euthanized as the humane endpoint. In some experiments, mice were weighed every two to three days, beginning at the time of adoptive transfer of the *ex vivo* activated T cells. For the survival experiments, we stopped the observation at 60–80 days after tumor inoculation, because deaths associated with tumor regrowth were rarely observed in mice that successfully eliminated tumors in several long-term observations.

### Isolation of tumor-infiltrating immune cells

The *ex vivo* activated CD4^+^ T cells were stained with carboxyfluorescein succinimidyl ester (CFSE) (Thermo Fisher Scientific, Waltham, MA, USA; # C34554) before intratumoral injection. The host mice were euthanized at 4 and 24 h after the immunotherapy, following which the tumor was resected and cut into small fragments (1–2 mm in width). The tumor fragments were incubated in RPMI-1640 medium containing 10 % FBS, collagenase type I (300 U/mL; Worthington Biochemical Corp., Lakewood, NJ, USA; # LS004196), and DNase I (0.1 mg/mL; Sigma-Aldrich; # DN25-100MG) at 37 °C for 120 min. The digested fragments were filtered using a 70-µm mesh, loaded over superimposed layers of 54 and 63 % Percoll (Cytiva, Marlborough, MA, USA; # 17,544,502), and centrifuged for 45 min at 400 × *g* at 20 °C. Tumor-infiltrating immune cells were recovered from the interface between the 54 and 63 % Percoll layers. These immune cells were used for further analyses and/or cell purification using a cell sorter. In some experiments, the tumor cells were recovered from the interface between the medium and the 54 % Percoll layer.

### Real-time RT-PCR

For gene expression analyses, due to the limited number of cells recovered from each mouse, we pooled samples from different mice (*n* = 3 for each group) within the same group before cell purification using a cell sorter. Total RNA was extracted from the sorted cells using TRIzol™ (Thermo Fisher Scientific; # 15,596,026). In some experiments, we extracted total RNA from the instantly frozen tumor samples by homogenization with TRIzol™ and liquid nitrogen. cDNA was generated using SuperScript™ IV VILO™ Master Mix with ezDNase™ Enzyme (Thermo Fisher Scientific; # 11,766,050) and quantified using quantitative real-time RT-PCR. Real-time PCR was performed using PowerUp™ SYBR™ Green PCR Mix (Thermo Fisher Scientific; # A25742) on a QuantStudio™ 6 Flex Real-Time PCR System (Thermo Fisher Scientific). Thermocycler conditions were as follows: initial holding at 50 °C for 2 min, then 95 °C for 2 min, followed by a three-step PCR program as follows: 95 °C for 15 s, 55 °C for 15 s, and 72 °C for 60 s for 40 cycles. Reactions were run in triplicate for each sample. Transcript abundance was calculated using the 2^−ΔΔCt^ method (normalization with 18 S or GAPDH). The primer sequences are listed in Supplementary Table S1.

### Immunohistochemistry and imaging

Ten-micrometer-thick frozen sections of the resected tumors were fixed in 100 % ethanol for 10 min at − 20 °C. After blocking with 5 % skimmed milk at room temperature for 30 min, the sections were incubated overnight at 4 °C with primary antibodies diluted with Signal Booster Immunostain Solution F (BCL-ISF; Beacle, Inc., Kyoto, Japan). They were rinsed again with PBS, followed by reaction for 1 h at room temperature with Cy3-conjugated streptavidin (AAT Bioquest, CA, USA; #16,912). In vitro activated donor cells were stained with Far Red (Thermo Fisher Scientific; # C34572) immediately before in vivo injection to detect the donor cells. All samples were examined using a laser-scanning confocal microscope (FV1000; Olympus, Tokyo, Japan). Photographs were processed using Photoshop CC (Adobe). Representative images obtained from three independent animals are shown.

### Statistical analysis

Survival rates among the different groups were compared using log-rank tests. Comparisons between two groups were performed using a two-tailed unpaired Student’s *t-*test. For multiple groups, the data were analyzed by ANOVA, followed by a posthoc Tukey’s multiple comparison test.

## Results

### AAA-CD4^+^ T cells can eliminate pre-established melanoma in mice

Similar to early clinical studies, our studies using Flt3L-DCs have demonstrated that the cancer vaccination of autologous DCs results in limited effects on a pre-established tumor [9, 12, 22–24] (Supplementary Fig. S1a, b). The pre-established tumor may develop an immunosuppressive environment to inhibit the induction of tumor-reactive T cells. To address this, we cultured B6 T cells together with B6 mouse-derived Flt3L-DCs to produce activated T cells. As a result, intratumoral injection of either autologous CD8^+^ T-cell melanoma antigen-activated by gp100-pulsed Flt3L-DCs or autologous CD4^+^ T cells activated by tumor lysate-pulsed Flt3L-DCs failed to mediate any measurable antitumor activity against pre-established B16F1 in B6 mice (Supplementary Fig. S2a-d). In this autologous setting, the administration of either DCs or DC-activated T cells was unable to induce antitumor activity in these tumor-bearing mice.

**Fig. 1:**
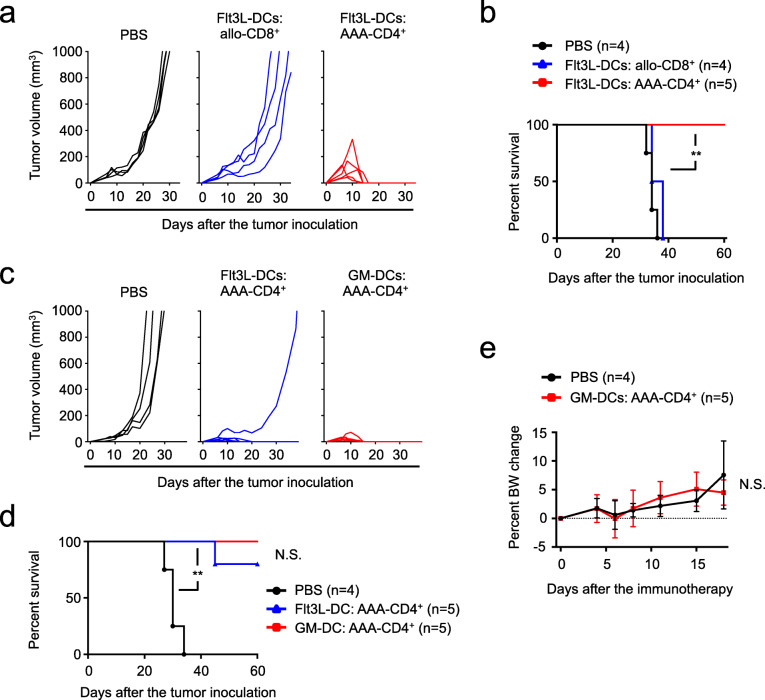
Alloantigen-activated CD4^+^ (AAA-CD4^+^) T cells can eliminate pre-established melanoma in mice. **a-b**: CD8^+^ and CD4^+^ T cells isolated from the spleens of BALB/c mice were separately activated in mixed lymphocyte cultures with B6-derived Flt3L-DCs pulsed with human gp100 peptides and the lysate of B16F1, respectively. Host B6 mice were subcutaneously injected with 1 × 10^6^ B16F1 cells on day zero. Nine days after B16F1 inoculation, the activated CD8^+^ T cells or activated CD4^+^ T cells (AAA-CD4^+^ T cells) were separately injected intratumorally into the host B6 mice. The PBS-injected group served as the control. Tumor growth (**a**) and survival (**b**) are shown. **c-e**: AAA-CD4^+^ T cells were produced from BALB/c mouse CD4^+^ T cells in cultures activated by either B6-derived Flt3L-DCs or GM-DCs. Host mice were subcutaneously injected with 1 × 10^6^ B16F1 cells on day zero. Nine days after B16F1 inoculation, AAA-CD4^+^ T cells were intratumorally injected. Tumor growth (**c**) and survival (**d**) are shown. Percent changes in BW of mice in the GM-DC-induced AAA-CD4^+^ and PBS-injected control groups are shown (**e**). Data are expressed as the mean ± SD. **P < 0.01. Abbreviations: N.S.: not significant

Allogeneic hematological malignant cells can be eliminated using infused donor T cells in allo-HSCT settings. This immune reaction is called the graft-versus-tumor (GVT) effect mediated by donor T cells that recognize and react to multiple alloantigens in tumor cells [44–48]. This beneficial GVT effect is initiated by host-derived DCs that activate allogeneic donor T cells [49, 50]. Given the potent GVT effect against hematological malignant cells, we hypothesized that alloreactive T cells could break the established immunosuppressive environment to elicit antitumor immunity in these melanoma-burdened mice. To that end, we stimulated BALB/c mouse-derived CD8^+^ T cells with B6 mouse-derived Flt3L-DCs pulsed with gp100. However, intratumoral injection of these activated allogeneic CD8^+^ T cells also failed to induce antitumor responses (Fig. 1a). In contrast, intratumoral injection of AAA-CD4^+^ T cells, which were produced from BALB/c mouse CD4^+^ T cells in B6 mouse-derived Flt3L-DCs pulsed with the tumor lysate elicited potent antitumor immunity against established B16F1. All mice treated with AAA-CD4^+^ T cells achieved complete tumor regression and survived for over 60 days without tumor recurrence (Fig. 1a-b). Interestingly, this result was reproduced by the AAA-CD4^+^ T cells that were activated in culture with the B6 mouse-derived GM-CSF induced conventional DCs (GM-DCs) (Fig. 1c-d, Supplementary Fig. S3). These results suggest that either Flt3L-DCs or GM-DCs enable AAA-CD4^+^ T cells to eliminate pre-established melanomas. Notably, without preparative conditioning and allo-HSCT, the mice that received AAA-CD4^+^ T cells did not show any clinical signs of GVHD, such as weight loss (Fig. 1e). Since GM-DC-based vaccines have been used in clinical trials for cancer treatment and large numbers of GM-DCs can be generated, we decided to use GM-DCs to induce AAA-CD4^+^ T cells for all subsequent experiments, unless mentioned otherwise.

### Anti-host alloreactivity is critical for AAA-CD4^+^ T cells to induce potent antitumor immunity

**Fig. 2:**
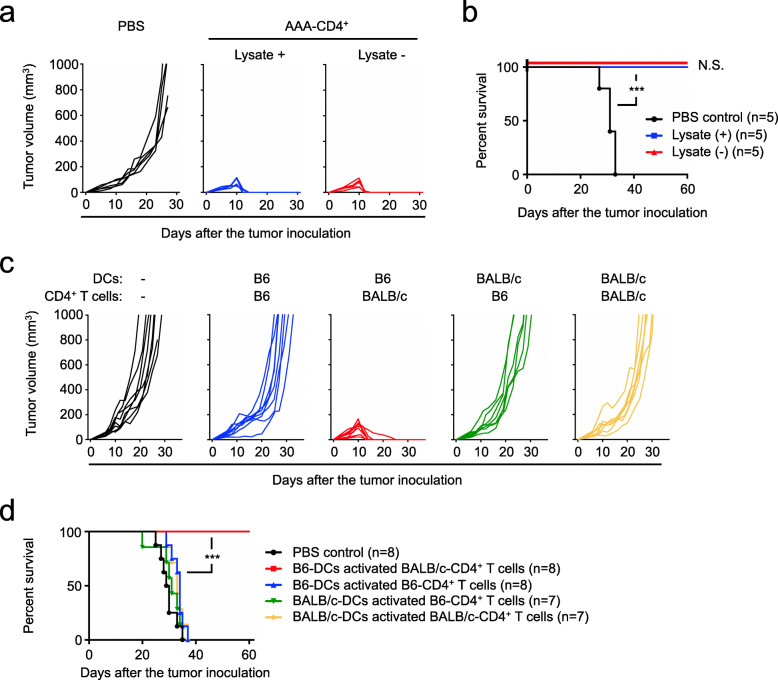
Anti-host alloreactivity is critical for AAA-CD4^+^ T cells to induce potent antitumor immunity. **a-b**: AAA-CD4^+^ T cells were produced from BALB/c mouse CD4^+^ T cells in cultures activated using B6-derived GM-DCs pulsed with or without B16F1 lysate. Host B6 mice were subcutaneously injected with 1 × 10^6^ B16F1 cells on day zero. Nine days after B16F1 inoculation, AAA-CD4^+^ T cells were intratumorally injected. Tumor growth (**a**) and survival (**b**) are shown. **c-d**: CD4^+^ T cells isolated from the spleens of B6 or BALB/c mice were separately activated in a mixed lymphocyte culture with activated B6 or BALB/c-derived DCs, as indicated. Host mice were subcutaneously injected with 0.5 × 10^6^ B16F1 cells on day zero. Nine days after B16F1 inoculation, activated CD4^+^ T cells were intratumorally injected into the host B6 mice. Tumor growth (**c**) and survival (**d**) are shown. The data are pooled from two independent experiments. ***P < 0.001. Abbreviations: N.S.: not significant

We next examined whether alloreactivity or tumor cell-derived antigens may be crucial for the induction of AAA-CD4^+^ T cells to destroy melanoma using two strategies. First, to test the impact of tumor cell-derived antigens, we pulsed GM-DCs with or without B16F1 tumor cell lysates and added them to BALB/c CD4^+^ T cell cultures. Irrespective of the tumor lysate stimulation, these B6 GM-DCs induced BALB/c AAA-CD4^+^ T cells with a potent capacity to eliminate B16F1 melanoma (Fig. 2a-b). Thus, tumor-associated antigens are unlikely to be required for the induction of tumor-destructive AAA-CD4^+^ T cells. Second, to assess whether alloreactive T cells specific to the B6 host are important for eliminating tumors, B6 CD4^+^ T cells were stimulated with BALB/c GM-DCs and injected into B16F1 melanoma cells. In this setting, the B6 CD4^+^ T cells were reactive to BALB/c DC-derived alloantigens but not the B6 host; they failed to inhibit tumor growth and progression (Fig. 2c-d). In addition, BALB/c CD4^+^ T cells activated using BALB/c DCs, which were not reactive to the B6 host, also failed to control tumor progression (Fig. 2c-d). In contrast, only host DC-induced AAA-CD4^+^ T cells acquired a unique ability to destroy pre-established melanoma (Fig. 2c-d). Furthermore, intratumoral injection of allogeneic naïve BALB/c CD4^+^ T cells or BALB/c CD4^+^ T cells that were non-specifically activated using CD3/CD28 antibody did not show any antitumor immunity in the same tumor model (Supplementary Fig. S4a-d). Collectively, these findings indicate that the beneficial antitumor activity of AAA-CD4^+^ T cells relies on their anti-host reactivity.

### Increased expansion and Th1-type inflammation of AAA-CD4^+^ T cells in the tumor post injection

**Fig. 3:**
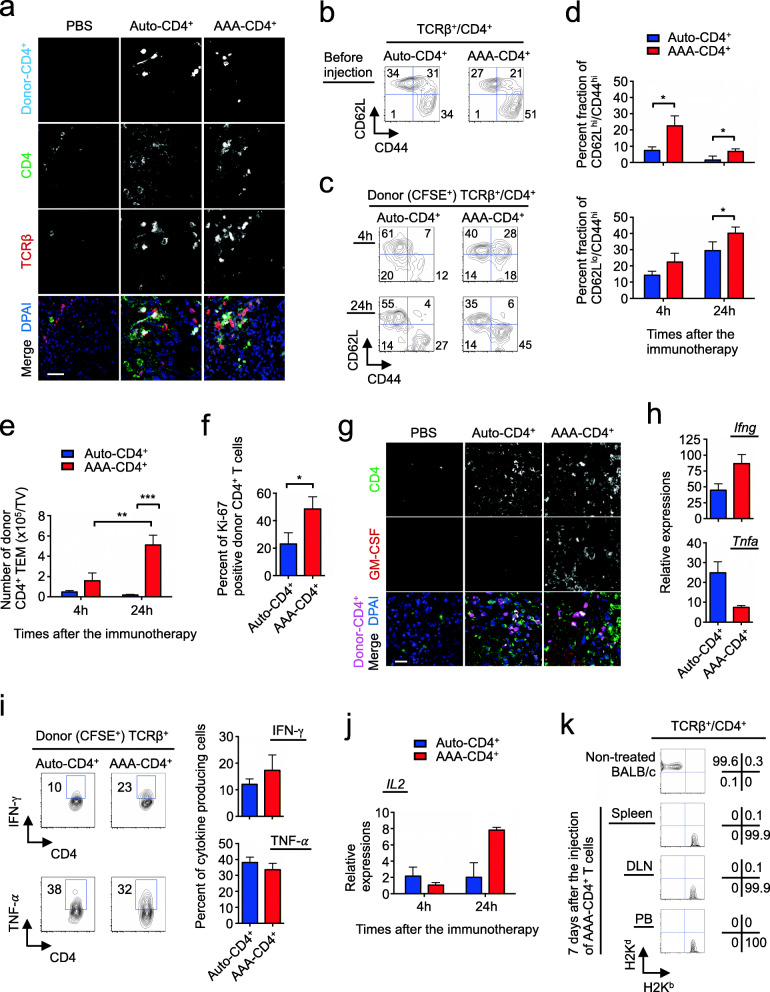
Increased expansion and Th1-type inflammation of AAA-CD4^+^ T cells in the tumor after injection. AAA-CD4^+^ T cells were produced from BALB/c mouse CD4^+^ T cells in cultures activated with B6-derived GM-DCs. Host B6 mice were subcutaneously injected with 1 × 10^6^ B16F1 cells on day zero. Nine days after B16F1 inoculation, AAA-CD4^+^ T cells that had been labeled with CFSE or Far Red were intratumorally injected (AAA-CD4^+^ group). B6-derived DC-activated B6-derived CD4^+^ T cells (auto-CD4^+^ group) and PBS-injected mice were used as controls (three mice per group). **a, g**: Representative confocal images of the indicated markers in tumors isolated 24 h after immunotherapy are shown. Bar, 20 μm. **b, c**: Representative scatterplots of the donor CD4^+^ T cells before *in vivo* injection (**b**) and those isolated from the tumors (**c**) are shown. **d, e**: The proportions of CD62L^hi^/CD44^hi^ central memory and CD62L^low^/CD44^hi^ effector memory populations (**d**) and the number of CD44^hi^/CD62L^low^ effector memory cells (**e**) in the donor CD4^+^ T cells isolated from the tumor. **f**: Graph shows the Intranuclear staining of Ki-67 in donor CD4^+^ T cells isolated from the tumor. **h, j**: Results show the relative expression levels of the indicated genes in the donor CD44^hi^/CD4^+^ T cells isolated from the tumors, normalized to those in naïve CD4^+^ T cells of non-treated BALB/c mice. **i**: Results show the Intracellular staining of indicated cytokines in donor CD4^+^ T cells isolated from the tumor and cultured for 10 h in a medium containing human IL-2 (200 IU/mL) with a protein transport inhibitor. **k**: Seven days after immunotherapy, peripheral blood, spleen, and draining lymph nodes were isolated. Representative scatterplots of chimerism analysis of donor CD4^+^ T cells are shown. Data are expressed as the mean ± SD. *P < 0.05, **P < 0.01, ***P < 0.001. Representative data from two independent experiments are shown. Abbreviations: TV: tumor volume

To understand the mechanism by which AAA-CD4^+^ T cells eliminate tumors, we first examined the distribution of donor cells in the tumor. Donor CD4^+^ T cells were labeled with CFSE or Far Red before injection to facilitate their detection in vivo. Confocal images showed both AAA-CD4^+^ T cells and auto-CD4^+^ T cells in the tumor of each treated mouse 24 h after injection (Fig. 3a). These donor cells were distributed unevenly in each tumor, and AAA-CD4^+^ T cells were observed in more areas compared to auto-CD4^+^ T cells. To determine these contents, we isolated tumor-infiltrating lymphocytes at 4 and 24 h after immunotherapy and performed FCM analysis (Supplementary Fig. S5). Before injection, approximately 50 % of the AAA-CD4^+^ T cells were CD44^hi^CD62L^lo^ effector memory T cells (TEM) (Fig. 3b). In previous studies, we have shown that these AAA-CD4^+^ T cells produce high levels of effector cytokines such as interferon gamma (IFN-γ) and tumor necrosis factor-α (TNF-α) [38]. Twenty-four hours after the intratumoral injection, a significantly higher frequency of CD4^+^ TEM cells was detected in mice receiving AAA-CD4^+^ T cells than in those receiving auto-CD4^+^ T cells (Fig. 3c,d). AAA-CD4^+^ TEM-cells underwent dramatic expansion (> 3-fold) within the tumor 24 h after injection relative to 4 h after injection. In contrast, the number of auto-CD4^+^ TEM-cells did not increase 24 h after injection. Consequently, there were 3-fold and 30-fold higher numbers of donor CD4^+^ TEM cells recovered from the tumors in mice that received AAA-CD4^+^ T cells compared to those that received auto-CD4^+^ T cells at 4 and 24 h after therapy, respectively (Fig. 3e). These intratumoral AAA-CD4^+^ T cells contained 2-fold Ki-67 positive cells compared to the auto-CD4^+^ T cells at 24 h after injection (Fig. 3f). Histologically, GM-CSF production was predominantly detected in the tumor isolated from AAA-CD4^+^ T cell-treated mice (Fig. 3 g). These data suggest that AAA-CD4^+^ T cells retain their proliferative capacity and the ability to produce GM-CSF, which is known to enhance anti-tumor immunity [51].

Real-time RT-PCR assays showed that CD44^hi^ donor CD4^+^ T cells in the AAA-CD4^+^ group produced 2-fold more IFN-γ transcripts but less TNF-α transcripts relative to that in the auto-CD4^+^ group at 4 h after injection (Fig. 3 h). We observed the same trend in the production of these cytokines at the protein level in the donor CD44^hi^ CD4^+^ T cells during in vitro culture after isolation from the tumor (Fig. 3i). Notably, the expression of IL-2 mRNA in the CD44^hi^ donor CD4^+^ T cells of the AAA-CD4^+^ group dramatically increased (7-fold) within the tumor 24 h after injection, relative to 4 h after injection, while IL-2 mRNA was not increased in the auto-CD4^+^ group (Fig. 3j). We also examined the persistence of injected donor CD4^+^ T cells in the host. Seven days after injection, donor-derived AAA-CD4^+^ T cells, which are H2Kd-positive CD4^+^ T cells, were not detected in the peripheral blood (PB), spleens, or draining lymph nodes (DLNs) of B6 mice that had eliminated the B16F1 melanoma (Fig. 3k). This suggests that AAA-CD4^+^ T cells themselves diminished during tumor regression by this time point, despite their induction of potent antitumor immunity in mice.

### AAA-CD4^+^ T cells license host endogenous CD8^+^ T cells to destroy melanoma

**Fig. 4:**
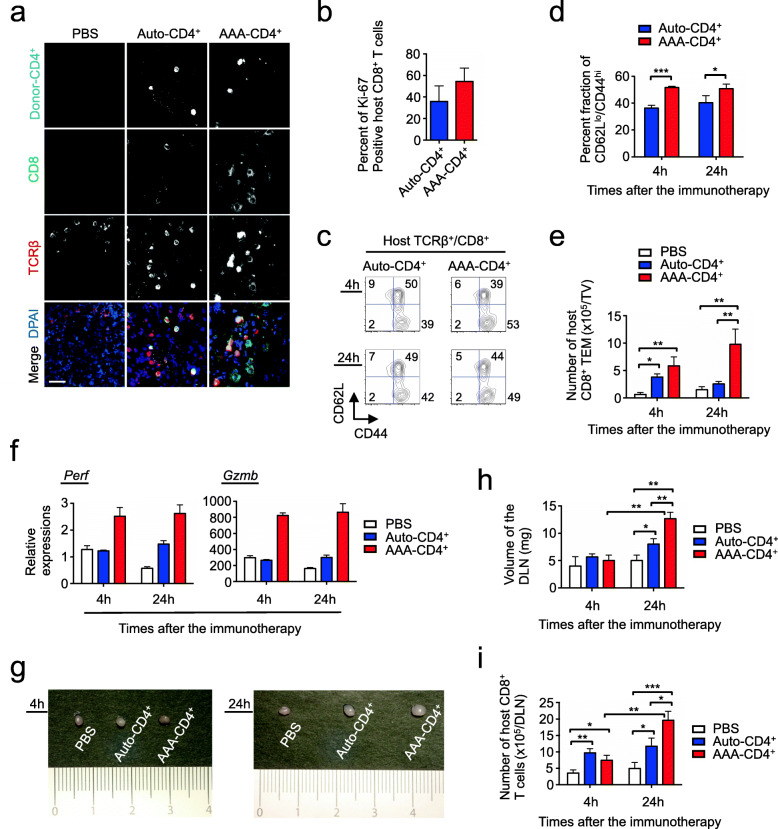
AAA-CD4^+^ T cells license endogenous CD8^+^ T cells to destroy melanoma. AAA-CD4^+^ T cells were produced from BALB/c mouse CD4^+^ T cells in cultures activated with B6-derived GM-DCs. Host B6 mice were subcutaneously injected with 1 × 10^6^ B16F1 cells on day zero. Nine days after B16F1 inoculation, AAA-CD4^+^ T cells that had been labeled with CFSE or Far Red were intratumorally injected (AAA-CD4^+^ group). B6-derived DC-activated B6-derived CD4^+^ T cells (auto-CD4^+^ group) and PBS-injected mice were used as controls (three mice per group). **a**: Representative confocal images of the indicated markers in tumors isolated 24 h after immunotherapy are shown. Bar, 20 μm. **b**: Graph shows the Intranuclear staining of Ki-67 in tumor-infiltrating host CD8^+^ T cells **c**: Representative scatterplots of tumor-infiltrating host CD8^+^ T cells. **d**: Results show the proportion of the CD62L^low^/CD44^hi^ effector memory population in tumor-infiltrating host CD8^+^ T cells. **e**: The number of host CD44^hi^/CD62L^low^ effector memory CD8^+^ T cells isolated from the tumor. **f**: Results show the relative expression levels of the indicated genes in the host CD44^hi^/CD8^+^ T cells isolated from the tumor, normalized to those in the host CD8^+^ T cells isolated from the spleen of PBS-treated mice. **g**: Representative photographs show macro-appearances of the DLNs at 4 and 24 h after the intratumoral injection of the *ex vivo* activated CD4^+^ T cells. **h**: Volumes of DLNs isolated from mice in each group are shown. **i**: The number of total host CD8^+^ T cells isolated from DLNs is shown. Data are expressed as the mean ± SD. *P < 0.05, **P < 0.01, ***P < 0.001. Representative data from two independent experiments are shown. Abbreviations: TV: tumor volume

To assess how AAA-CD4^+^ T cells elicit antitumor responses, we analyzed the host immune cells that infiltrated the tumor. Confocal images showed that tumor-infiltrating host CD8^+^ T cells were observed in more areas in the tumor from AAA-CD4^+^ T cell-treated mice than in auto-CD4^+^ T cell-treated mice (Fig. 4a). FCM analysis revealed that about half of the tumor-infiltrating host CD8^+^ T cells were positive for Ki-67 in AAA-CD4^+^ T cell-treated mice. These CD8^+^ T cells expressed both CD44^hi^CD62L^lo^ TEM-cells and CD44^hi^CD62L^hi^ central memory T cells (TCM) phenotypes (Fig. 4b,c). There was a significantly higher frequency of host CD8^+^ TEM-cells in the AAA-CD4^+^ group than in the auto-CD4^+^ group at 4 and 24 h after injection (Fig. 4d). Intratumoral injection of AAA-CD4^+^ T cells recruited 10-fold and 7-fold more tumor-infiltrating host CD8^+^ TEM cells at 4 and 24 h after injection, respectively, compared to the PBS-treated control mice (Fig. 4e). Although intratumoral injection of activated auto-CD4^+^ T cells also led to increased infiltration of host CD8^+^ TEM cells at 4 h after injection, additional enrichment was not observed at 24 h after injection (Fig. 4e). As a result, AAA-CD4^+^ T cells induced approximately 4-fold more host CD8^+^ TEM cells in the tumor 24 h after injection, compared to auto-CD4^+^ T cells (Fig. 4e). Intriguingly, host CD44^hi^CD8^+^ T cells that were recruited by AAA-CD4^+^ T cells produced higher levels of perforin and granzyme B than those recruited by auto-CD4^+^ T cells (Fig. 4f). Thus, these tumor-infiltrating host CD8^+^ T cells derived from AAA-CD4^+^ T cell-treated mice showed a high cytolytic capacity.

Intra-tumoral injection of AAA-CD4^+^ T cells also induced significantly more host CD44^hi^CD62L^lo^ CD4^+^ TEM cells 24 h after injection compared to auto-CD4^+^ T cells (Supplementary Fig. S6a-b). These host CD4^+^ T cells derived from AAA-CD4^+^ T cell-treated mice produced higher levels of *Ifng* compared to those from auto-CD4^+^ T cell-treated mice and PBS-treated control mice at 24 h (Supplementary Fig. S6c). These data suggest that injection of AAA-CD4^+^ T cells also leads to the activation of host CD4^+^ T cells that produce IFN-γ.

Twenty-four hours after injection, AAA-CD4^+^ T cell-treated mice displayed a marked increase in the overall size of the DLN and numbers of both host CD8^+^ and CD4^+^ T cells in the DLN, compared to the auto-CD4^+^ T cell-treated mice and PBS-treated control mice (Fig. 4g-i; Supplementary Fig. S6d). These results indicate that AAA-CD4^+^ T-cell therapy leads to significant expansion of both host CD8^+^ and CD4^+^ T cells in the tumor and DLN.

### Host endogenous CD8^+^ T cells and CD4^+^ T cells are required to mediate antitumor immunity in mice undergoing AAA-CD4^+^ T-cell therapy

**Fig. 5:**
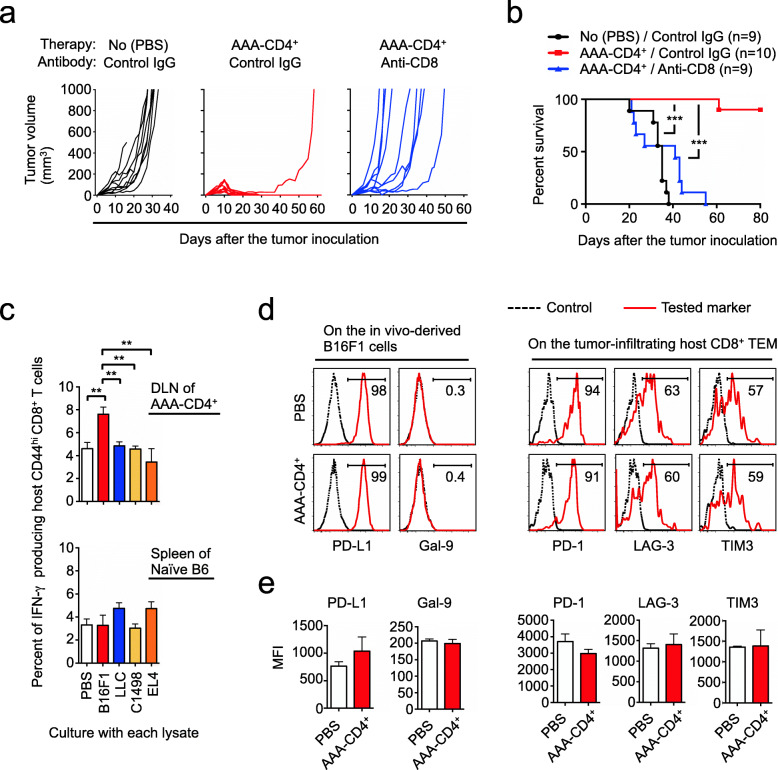
Host endogenous CD8^+^ T cells are required to mediate antitumor immunity in AAA-CD4^+^ T-cell therapy. AAA-CD4^+^ T cells were produced from BALB/c mouse CD4^+^ T cells in cultures activated with B6-derived GM-DCs. Host B6 mice were subcutaneously injected with 1 × 10^6^ B16F1 cells on day zero. Nine days after B16F1 inoculation, AAA-CD4^+^ T cells were intratumorally injected. **a-b**: Before injection of the AAA-CD4^+^ T cells, an anti-mouse CD8-eliminating antibody or control IgG was intraperitoneally injected at a dose of 500 µg on day 8, and 250 µg on days 10 and 12. Tumor growth (**a**) and survival (**b**) are shown. The data are pooled from two independent experiments. **c**: Twenty-four hours after the intratumoral injection of AAA-CD4^+^ T cells, the host mice were euthanized, and draining lymph nodes (DLNs) were resected (three mice per group). After making a single-cell suspension of DLNs, CD8^+^ T cells were isolated and cultured in a medium containing human IL-2 (200 IU/mL) with lysates prepared from several B6-origin tumor cell lines. After 4 h of incubation with a protein transport inhibitor, the cells were recovered, stained with intracellular IFN-γ, and analyzed using flow cytometry. CD8^+^ T cells isolated from the spleens of naïve B6 mice were used as controls (three mice per group). **d-e**: Twenty-four hours after the intratumoral injection of AAA-CD4^+^ T cells, the host mice were euthanized and tumors were resected. Subsequently, the tumor cells and tumor-infiltrating mononuclear cells were isolated. Representative histograms (**d**) and mean fluorescent intensities (**e**) show the indicated immune checkpoint molecules on the surface of either *in vivo* isolated tumors or tumor-infiltrating host CD8^+^ TEM cells. Data are expressed as the mean ± SD. **P < 0.01, ***P < 0.001. Representative data from two independent experiments are shown

To examine the precise role of host tumor-infiltrating lymphocytes in the AAA-CD4^+^ T cell-mediated elimination of melanoma, we first depleted host CD8^+^ T cells using an antibody specific to CD8 before injection of AAA-CD4^+^ T cells. Administration of control IgG did not impair the antitumor effects of the AAA-CD4^+^ T-cell therapy (except for one sample that failed to develop antitumor immunity) (Fig. 5a-b). In contrast, depleting host CD8^+^ T cells abrogated the antitumor activity of AAA-CD4^+^ T cells, with all mice succumbing to the tumor by day 55 (Fig. 5a-b). These findings suggest that AAA-CD4^+^ T cells have a unique ability to license endogenous CD8^+^ cytotoxic T-lymphocytes (CTLs) to destroy tumor cells. To assess the specificity of these host CD8^+^ T cells against B16F1 melanoma, we isolated host CD8^+^ T cells from DLNs 24 h after AAA-CD4^+^ T-cell therapy and cultured them in a medium containing tumor cell lysates derived from a variety of B6 mouse tumor cell lines, including B16F1, Lewis lung carcinoma, C1498 leukemia, and EL4 lymphoma cells (Fig. 5c). These host CD44^hi^CD8^+^ T cells produced significantly higher amounts of IFN-γ upon co-culture with B16F1 lysates compared to non-relevant tumor cell lysates (Fig. 5c), confirming the specificity of host CD8^+^ T cells against B16F1 cells.

Next, we examined the importance of host CD4^+^ T cells using B6 background CD4 knockout mice as hosts. Although some CD4 knockout mice showed delayed tumor growth after AAA-CD4^+^ T-cell therapy, all mice succumbed to tumor growth by day 51 (Supplementary Fig. S7a-b). This suggests that the host CD4^+^ T cells also play critical roles in the induction of anti-tumor immunity by AAA-CD4^+^ T-cell therapy.

NK cells are innate immune cells that can kill tumor cells [52]. Although both AAA-CD4^+^ and auto-CD4^+^ T-cell therapies induced NK cell infiltration in the tumor at 4 h, this effect was maintained only in the AAA-CD4^+^ group at 24 h (Supplementary Fig. S8a). As a result, 24 h after intratumoral injection, mice that received AAA-CD4^+^ T-cell therapy had significantly more host-type NK cells in the tumor than mice that received auto-CD4^+^ T cells and PBS-treated control mice (Supplementary Fig. S8a). However, depletion of NK cells prior to AAA-CD4^+^ T-cell therapy did not impair the elimination of melanoma (Supplementary Fig. S8b-c). Thus, NK cells are unlikely to mediate the antitumor activity of AAA-CD4^+^ T-cell therapy. We also examined the kinetics of host B cells in the tumor after AAA-CD4^+^ T-cell therapy, which showed no statistical difference among the three groups at 4 and 24 h after immunotherapy (Supplementary Fig. S9).

**Fig. 6:**
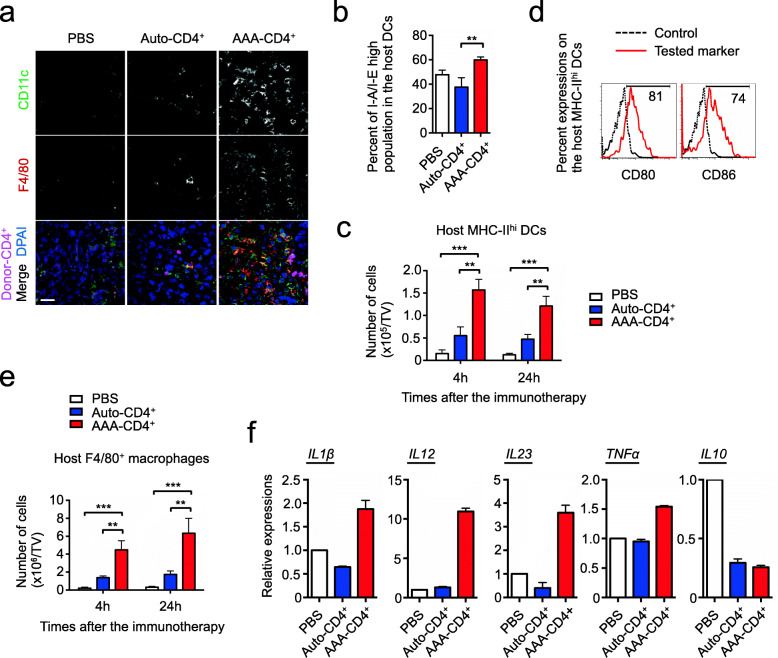
AAA-CD4^+^ T-cell therapy increases tumor infiltration of host-activated DCs and activated macrophages. AAA-CD4^+^ T cells were produced from BALB/c mouse CD4^+^ T cells in cultures activated with B6-derived GM-DCs. Host B6 mice were subcutaneously injected with 1 × 10^6^ B16F1 cells on day zero. Nine days after B16F1 inoculation, AAA-CD4^+^ T cells were intratumorally injected (AAA-CD4^+^ group). B6-derived DC-activated B6-derived CD4^+^ T cells (auto-CD4^+^ group) and PBS-injected mice were used as controls (three mice per group). **a**: Representative confocal images of the indicated markers in tumors isolated 24 h after immunotherapy are shown. Bar, 20 μm. **b**: Graph shows the percentage of I-A/I-E high proportion of host CD11c^+^/F4/80^−^DCs at 4 h after therapy. **c**: Graph showing the number of host CD11c^+^/MHC-II^hi^/F4/80^−^ DCs that infiltrated the tumor. **d**: Representative histogram showing the percentage expression of the indicated co-stimulatory molecules on the host CD11c^+^/MHC-II^hi^/F4/80^−^ DCs in the AAA-CD4^+^ T cell group at 24 h after therapy. **e**: The number of host F4/80^+^ macrophages that infiltrated the tumor is shown. **f**: Relative expression levels of the indicated genes in the tumor-infiltrated host F4/80^+^ macrophages in each group at 24 h after therapy, normalized to those in PBS-treated mice. Data are expressed as the mean ± SD. **P < 0.01, ***P < 0.001. Representative data from two independent experiments are shown. Abbreviations: TV: tumor volume

We further examined immune checkpoint changes during the AAA-CD4^+^ T cell-induced immune response. First, we analyzed the expression of immune checkpoint ligands on the surface of the in vivo isolated B16F1 tumor. As a result, most of the tumor cells expressed high levels of PD-L1 but not galectin-9 (Gal-9, a ligand for Tim3) (Fig. 5d-e). There were no statistical differences in the mean fluorescence intensities (MFI) of these ligands between PBS-treated control mice and AAA-CD4^+^ T cell-treated mice (Fig. 6e). Next, we examined the expression of immune checkpoint receptors on the surface of tumor-infiltrating host CD8^+^ TEM cells. More than 90 % of tumor-infiltrating host CD8^+^ TEM cells expressed PD-1, while approximately 60 % expressed LAG-3 and/or TIM3 (Fig. 5d-e). There were no statistical differences in the MFI of these receptors on the surface of host CD8 TEM cells between PBS-treated control mice and AAA-CD4^+^ T cell-treated mice (Fig. 5d-e). Given the potent anti-tumor activity of AAA-CD4^+^ T cells (Figs. 1 and 2), these results indicate that the therapeutic effect of AAA-CD4^+^ T cells is likely independent of these inhibitory receptors.

### AAA-CD4^+^ T-cell therapy increases tumor infiltration of host-activated DCs and activated macrophages

To understand the mechanism by which AAA-CD4^+^ T cells license host endogenous CD8^+^ T cells to eliminate melanoma, we examined the potential effects of other immune cells in tumors and DLNs. Confocal images showed a significant increase in host CD11c-positive DCs infiltrated in the tumor from AAA-CD4^+^ T cell-treated mice compared to that in PBS control mice and auto-CD4^+^ T cell-treated mice (Fig. 6a). DCs are one of the most important professional antigen-presenting cells (APCs) that activate T cells [53, 54]. The isolated tumor-infiltrating DCs in mice that received AAA-CD4^+^ T-cell therapy showed significantly higher expression of MHC class II molecules, compared to mice in the auto-CD4^+^ T cell group at 4 h after therapy (Fig. 6b). There were significantly more MHC class II^high^ DCs that infiltrated into the tumor in mice treated with AAA-CD4^+^ T cells as early as 4 h after therapy, as compared to control mice (10-fold) and auto-CD4^+^ T cell-treated mice (3-fold) (Fig. 6c). This relationship was maintained for 24 h following AAA-CD4^+^ T-cell therapy (Fig. 6c). These tumor-infiltrating MHC class II^high^ DCs derived from AAA-CD4^+^ T cell-treated mice expressed high levels of CD80 and CD86 on their surface (Fig. 6d). Additionally, AAA-CD4^+^ T-cell therapy also induced significantly more F4/80-positive host macrophages at 24 h after therapy compared to the control (20-fold) and auto-CD4^+^ T cell (3-fold) treatments (Fig. 6a,e). RT-PCR analysis revealed that these tumor-infiltrating macrophages expressed higher levels of transcripts encoding IL-1β, IL-12, IL-23, and TNF-α in the AAA-CD4^+^ group than in the control or auto-CD4^+^ groups (Fig. 6f). Interestingly, there was a marked decrease in IL-10 transcript levels in macrophages derived from mice treated with AAA-CD4^+^ T cells compared to PBS-treated control mice (Fig. 6f). We further confirmed that AAA-CD4^+^ T-cell therapy led to a significantly increased number of activated host DCs and macrophages in the DLNs (Supplementary Fig. S10a-c). These results suggest that both tumor-infiltrating host DCs and M1-like macrophages are recruited and activated upon AAA-CD4^+^ T-cell treatment. These APCs may play an important role in the regulation of AAA-CD4^+^ T cells by licensing endogenous CD8^+^ CTLs to eradicate tumors.

Regulatory T cells (Tregs) are known to regulate both T-cell expansion and Th1 response [55]. We examined the host CD4^+^/CD25^+^/FoxP3^+^ Tregs after AAA-CD4^+^ T-cell therapy. The number of Tregs in the tumor and peripheral blood of the AAA-CD4^+^ T cell-treated mice showed a slight decrease, but no statistical difference compared to that of the PBS-treated control mice. These data suggest that AAA-CD4^+^ T cell-therapeutic effects are likely to be achieved without reducing Tregs (Supplementary Fig. S11).

We also examined the effect of AAA-CD4^+^ T cells on the tumor microenvironment. Real-time RT-PCR analysis showed that the tumors isolated from AAA-CD4^+^ T cell-treated mice produced significantly higher levels of genes encoding immunosuppressive biomarkers, such as arginase 1 (ARG1), inducible nitric oxide synthase (iNOS), and cyclooxygenase-2 (COX2), compared to tumors derived from PBS-injected control mice (Supplementary Fig. S12). These data suggest that despite the presence of these immunosuppressive molecules within the tumor, intratumoral injection of AAA-CD4^+^ T cells retained their ability to elicit host endogenous antitumor immunity.

### Local AAA-CD4^+^ T-cell therapy elicits systemic antitumor activity to eliminate melanoma growth at distal sites

**Fig. 7:**
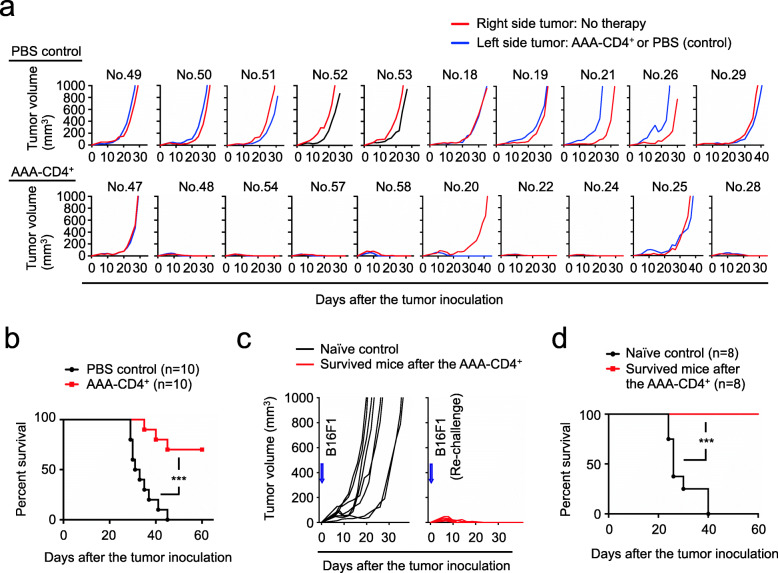
AAA-CD4^+^ T-cell therapy induces systemic and persistent antitumor immunity. AAA-CD4^+^ T cells were produced from BALB/c mouse CD4^+^ T cells in cultures activated with B6-derived GM-DCs. **a-b**: Host B6 mice were inoculated with the same amount of B16F1 cells (1 × 10^6^) on the left and right flanks of the mice on day zero. AAA-CD4^+^ T cells were intratumorally injected into the left side of the tumor. PBS-injected mice were used as controls. Tumor growth on both sides of each mouse (**a**) and survival (**b**) are shown. The data are pooled from two independent experiments. **c-d**: Nine days after inoculation with 0.5 × 10^6^ B16F1 cells, AAA-CD4^+^ T cells were intratumorally injected into the host B6 mice. The mice that survived AAA-CD4^+^ T-cell therapy were maintained for 6 months. Subsequently, the surviving mice were subcutaneously injected with 2.5 × 10^6^ B16F1 cells as a re-challenge for the same tumor. Naïve B6 mice were used as controls. Tumor growth (**c**) and survival (**d**) are shown. ***P < 0.001. The data are pooled from two independent experiments

In the next step of the study, we tested whether our current strategy induced local or systemic antitumor immune responses. For this, we inoculated B16F1 tumors on the left and right flanks of the mice and injected AAA-CD4^+^ T cells into the tumor on the left side. We found that AAA-CD4^+^ T-cell therapy induced tumor regression on both sides in 7 out of 8 mice (Fig. 7a). Two mice that failed to elicit antitumor activity against the tumor injected with AAA-CD4^+^ T cells also failed to control tumor growth on the other side (Fig. 7a). As a result, 7 out of 10 mice survived AAA-CD4^+^ T-cell therapy (Fig. 7b). These observations suggest that intratumoral injection of AAA-CD4^+^ T cells induced antitumor immunity, not only at the injected site but also at distal sites where AAA-CD4^+^ T cells were not directly administered. Thus, local delivery of AAA-CD4^+^ T cells triggers systemic antitumor immunity.

### AAA-CD4^+^ T-cell therapy induces persistent antitumor immunity in mice

Finally, we examined whether AAA-CD4^+^ T cell-induced host CTLs against B16F1 melanoma provided persistent protection against tumor recurrence in these mice. Six months after the initial B16F1 inoculation, we found that the B6 tumor mice that survived after AAA-CD4^+^ T-cell therapy resisted the B16F1-re-challenge (Fig. 7c-d). Thus, intratumoral injection of AAA-CD4^+^ T cells induces long-term protection against B16F1 melanoma and prevents tumor recurrence.

## Discussion

It has been shown that the mechanisms of tumor escape preclude the development of an effective antitumor immune response [56]. To escape from host immunity, tumor cells may develop a tumor microenvironment that has a suppressive effect on the immune system and causes tolerance of tumor-reactive T cells [56, 57]. Developing approaches capable of breaking this escape in tumor-bearing hosts has proven to be a major challenge. In the present study, we report the development of a novel and clinically relevant approach in which AAA-CD4^+^ T cells are able to license endogenous tumor-reactive CD8^+^ T cells to destroy tumors, leading to significantly improved overall survival rates in tumor-bearing animals.

Allogeneic CD4^+^ T cells have a unique potential to activate host immunity; however, the underlying mechanism is yet to be defined. In the present study, compared to auto-CD4^+^ T cells, intratumorally injected AAA-CD4^+^ T cells significantly expanded in the tumor and produced high levels of IFN-γ as early as 4 h post-therapy, which may induce initial immunogenic cancer cell death. The AAA-CD4^+^ T cells also recruited significantly more host DCs and macrophages into the tumor, which expressed high levels of antigen-presenting molecules (MHC class II), co-stimulatory molecules (e.g., CD80 and CD86), and cytokines (IL-12 and IL-23). All these molecules are known to be critical for the proliferation, survival, and effector functions of antigen-activated T cells. Recent studies have demonstrated that IL-12 and IFN-γ are critical for local APCs to activate tumor-reactive T cells to disrupt tumor escape in the host treated with anti-PD-1 antibody [58]. In addition, compared to auto-CD4^+^ T cells, AAA-CD4^+^ T cells produced 4-fold higher amounts of IL-2 transcripts at 24 h post-therapy, which led to more than a 100-fold higher concentration of IL-2 in the local tumor. IL-2 signals have been known to be critical for effector differentiation as well as the formation of long-lasting CD8^+^ T cell memory [59, 60]. These results may explain the observation that AAA-CD4^+^ T cells led to dramatically higher tumor-reactive host CD8^+^ CTLs in the tumor and DLNs at 24 h post-injection.

In addition to destroying the locally treated tumor, AAA-CD4^+^ T-cell therapy also induced systemic immune responses that eliminated the non-treated tumor located in a distant location. This is important for the effective control of metastatic tumors. Since locally administered AAA-CD4^+^ T cells were not tumor-specific antigen-reactive and were not detected in the spleen 7 days after treatment, it is unlikely that AAA-CD4^+^ T cells migrated to the distant tumor to elicit potent antitumor immunity. One plausible explanation is that the AAA-CD4^+^ T cell-licensed endogenous tumor-specific CD8^+^ T cells may circulate in the host and react with the cognate tumor-specific antigen on distant tumor cells. Further studies will be required to test this possibility and investigate the underlying mechanism by which AAA-CD4^+^ T-cell therapy elicits systemic immune responses.

Tumor cells can evade immune elimination through complex mechanisms, such as loss of antigenicity and impairment of immunogenicity [54, 59, 61]. The induction of an immunosuppressive microenvironment represents another important mechanism by which tumors suppress T cell immunity [56]. For example, ARG1, which may be produced by tumor-infiltrating myeloid cells such as myeloid-derived suppressor cells and macrophages, is a potent inhibitor of T cell responses [62–64]. iNOS is associated with diverse functions in inflammatory responses and T-cell suppression effects [65]. COX2 expression is known to increase the abundance of the enzymatic product prostaglandin E2 (PGE2) [66]. PGE2 has been shown to induce cancer cell migration and mediate immune suppression in a context-dependent manner [67, 68]. We found that although AAA-CD4^+^ T-cell therapy increased the expression of genes encoding ARG1, iNOS, and COX2 in the tumor, it retained the ability to destroy established tumors in mice. Future studies will investigate the mechanism by which AAA-CD4^+^ T cells overcome environmental immunosuppression to eliminate tumors.

Our study has important translational implications. For example, AAA-CD4^+^ T cells can be generated from a healthy donor upon stimulation with DCs derived from a patient with cancer. Intratumoral injection of these host DC-induced healthy donor AAA-CD4^+^ T cells may induce antitumor effects *in vivo*. One of the keys to induce the therapeutic effects of AAA-CD4^+^ T cells is an allogeneic immune reaction that triggers inflammation in the tumor. Therefore, DCs that express alloantigens of the tumor-bearing host must be used for the activation of allogeneic CD4^+^ T cells. Because CD4^+^ T cells are activated through MHC class II molecules, DCs from a third party that share MHC class II molecules with a tumor-bearing host should also be available to generate the intended AAA-CD4^+^ T cells (Supplementary Fig. S13). It has been reported that a large number of DCs can be generated from human cord blood; therefore, human AAA-CD4^+^ T cells can be generated and stocked in advance by using cord blood-derived DCs and allogeneic CD4^+^ T cells from healthy donors [69, 70]. This protocol would allow us to obtain and administer an appropriate AAA-CD4^+^ T cell product soon after an indication based on the MHC type of the patient, which is similar to regular blood transfusion.

There may be a concern about the possibility of inducing GVHD-like adverse effects on the use of host-reactive T cells. In fact, none of the mice that received AAA-CD4^+^ T cells showed immune-related complications or undesired adverse effects. These AAA-CD4^+^ T cells secrete high levels of IFN-γ, which induces inflammatory reactions in the tumor. Our previous study suggested that intravenous injection of these activated allogeneic CD4^+^ T cells failed to mediate severe acute GVHD in mouse models undergoing allogeneic HSCT [38]. These highly activated host-reactive donor T cells showed a decreased ability to survive for a long time following adoptive transfer, as they undergo apoptotic death *in vivo* [38]. In addition, they showed a reduced capability of migrating to GVHD target organs due to the downregulation of trafficking-related molecules [38]. These observations coincide with our findings, wherein we did not detect AAA-CD4^+^ T cells in the PB, DLNs, or spleens at 7 days after administration. Thus, the intrinsic program in AAA-CD4^+^ T cells may be the primary mechanism responsible for their inability to mediate GVHD. Furthermore, the lack of preparative conditioning in these tumor-bearing mice, which is known to be critical for sustaining donor cell engraftment and eliciting GVHD, may also contribute to the inability of AAA-CD4^+^ T cells to induce damage to the host tissues [44–46].

Recent clinical studies have demonstrated that HLA-mismatched granulocyte colony-stimulating factor–mobilized donor peripheral blood stem cell (G-PBSC) infusion after regular chemotherapy improves the outcome in elderly patients with acute myeloid leukemia (AML) [71–74]. Although the recipients did not receive any GVHD prophylaxis, GVHD was rarely observed. Interestingly, a significant increase in WT1^+^CD8^+^ T cells was observed after the microtransplantation, which was correlated with better survival rates [73]. These observations indicate that microtransplantation may be an effective and safe therapy for older patients with AML. In our current study, priming AAA-CD4^+^ T cells with host antigen-expressing DCs enabled them to preferentially react with host alloantigens. Therefore, AAA-CD4^+^ T cells start to induce an allogeneic immune reaction early after intratumoral injection. Subsequently, a strong inflammation triggered by the AAA-CD4^+^ T cells in the tumor reinvigorated host immunity, which plays a critical role in eradicating pre-established tumors. Importantly, the induced host tumor-specific CTLs persisted long-term in vivo to protect against tumor recurrence. Collectively, data from our studies and others [71–74] indicate that allogeneic cells have the potential to induce host anti-tumor immunity and may induce long-term protection against both solid tumors and AML.

We propose that a combination therapy based on alloantigen-activated CD4^+^ T cells may prove to be an effective strategy. The current AAA-CD4^+^ T-cell therapy is distinct from other anti-cancer therapies, such as chemotherapy, radiation therapy, and most other immune therapies. Hence, all these preexisting therapies can be performed together with AAA-CD4^+^ T-cell therapy, which potentially elicits an additive anti-cancer effect. Intriguingly, because the AAA-CD4^+^ T cells accelerate anti-cancer immune responses, combination therapy with one of the inhibitors of immune suppression, such as immune checkpoint blockade therapies can be further effective in inducing anti-tumor immunity.

One of the limitations that could affect the efficacy of the current approach is related to the loss or downregulation of MHC class I antigens by tumor cells, which is a frequent mechanism of immune escape [56, 57, 61]. If tumor cells completely lose the MHC class I gene, it may be difficult to achieve the therapeutic benefit of AAA-CD4^+^ T-cell therapy. In other cases, expression of MHC class I molecules on tumor cells may be rapidly induced by IFN-γ derived from Th1 cell responses triggered by AAA-CD4^+^ T cells. Some issues remain unaddressed in this study. In the process of tumor destruction, although the host antitumor CTLs are critical to eliminating the pre-established melanoma, the relative contributions of other immune cells in destroying the tumor are yet to be determined. Future studies could investigate what fraction of tumor destruction is due to the AAA-CD4^+^ T cells and what is due to the host immune cells, including CD8^+^ CTLs. It will also be interesting to identify which tumor-associated antigens are targeted by AAA-CD4^+^ T cell-licensed host CD8^+^ CTLs in tumor-bearing mice. We observed that CD8^+^ CTLs isolated from DLNs early after AAA-CD4^+^ T-cell therapy produced high levels of IFN-γ upon re-encounter with the tumor lysate. Therefore, we propose that our strategy may allow for the discovery of neoantigen-specific CD8^+^ T cells that can be utilized to augment antitumor immunity.

## Conclusions

We established a novel and clinically relevant cellular therapy using AAA-CD4^+^ T cells to induce potent endogenous antitumor immunity that eliminated pre-established melanoma without any complications. The reinvigorated host antitumor immunity persisted for a long time *in vivo* and provided long-term protection against tumor recurrence. Furthermore, local delivery of AAA-CD4^+^ T cells induces systemic antitumor immune responses against distant tumors. This suggests that our newly developed strategy may be potentially applied to the treatment of patients with metastatic diseases. This approach can be immediately translated into patients with advanced melanoma and may have broad implications for the treatment of other types of solid tumors.

## Supplementary Information

**Additional file 1: Figure S1.** Intratumoral injection of activated DCs failed to induce antitumor immunity to eliminate pre-established melanoma. **Figure S2. **Intratumoral injection of *ex vivo* activated autologous T cells failed to induce antitumor immunity to eliminate pre-established melanoma.  **Figure S3.** Representative photographs of tumor growths after the AAA-CD4^+^ T-cell therapy. **Figure S4. **Intratumoral injection of allogeneic naïve CD4^+^ T cells and CD3/CD28-Ab-activated allogeneic CD4^+^ T cells failed to induce antitumor immunity to eliminate pre-established melanoma. **Figure S5. **Representative scatterplots to detect specific populations of tumor-infiltrating T cells.  **Figure S6.** Increased infiltration and Th1-type inflammation of the host CD4^+^ T cells after AAA-CD4^+^ T-cell therapy.  **Figure S7. **Host endogenous CD4^+^ T cells are required to mediate antitumor immunity in mice undergoing AAA-CD4^+^ T-cell therapy.  **Figure S8. **Tumor-infiltrating host NK cells are not essential to induce antitumor immunity in AAA-CD4^+^ T-cell therapy.  **Figure S9. **The number of tumor-infiltrating host B cells after the AAA-CD4^+^ T-cell therapy.  **Figure S10. **Larger number of host-activated DCs and macrophages were isolated from the draining lymph nodes of the AAA-CD4^+^group, as compared to auto-CD4^+^ and control groups at 24 h after therapy. **Figure S11.** The number of host Tregs in the tumor and peripheral blood after the AAA-CD4^+^ T-cell therapy.  **Figure S12. **Gene expressions of immunosuppressive biomarkers in the tumor after the AAA-CD4^+^ T-cell therapy. **Figure S13. **DCs generated from 129X1 mice that share MHC class II molecules with B6 mice induced AAA-CD4^+^ T cells elicited antitumor immunity in the B16F1 inoculated B6 mice. **Table S1. **Primers for real-time RT-PCR.

## Data Availability

The datasets used and/or analyzed during the current study are available from the corresponding author upon reasonable request.

## Abbreviations

AAA: Alloantigen-activated
AML: Acute myeloid leukemia
APCs: Antigen-presenting cells
ARG1: Arginase 1
B6: C57BL/6
BM: Bone marrow
BW: Body weight
CD: Cluster of differentiation
CFSE: Carboxyfluorescein succinimidyl ester
COX2: Cyclooxygenase-2
CTLs: Cytotoxic T-lymphocytes
DCs: Dendritic cells
DLNs: Draining lymph nodes
FCM: Flow cytometry
Flt3L: FMS-like tyrosine kinase 3 ligand
Flt3L-DCs: Flt3L-induced DCs
Gal-9: Galectin-9
GM-CSF: Granulocyte macrophage colony-stimulating factor
GM-DCs: GM-CSF-induced DCs
GVHD: Graft-versus-host disease
GVT: Graft-versus-tumor
*Gzmb*: Granzyme B
HSCT: Hematopoietic stem cell transplantation
IDO: Indoleamine 2,3-dioxygenase
IFN-γ: Interferon gamma
IL: Interleukin
iNOS: Inducible nitric oxide synthase
LAG-3: Lymphocyte activation gene 3
LPS: Lipopolysaccharide
MFI: Mean fluorescent intensity
MHC: Major histocompatibility complex
NK: Natural killer
N.S.: Not significant
PB: Peripheral blood
PD-1: Programmed cell death-1
PD-L1: Programmed cell death ligand-1
Prf: Perforin
SCF: Stem cell factor
TCM: Central memory T cells
TEM: Effector memory T cells
Th1: T helper-1
TIM3: T cell immunoglobulin and mucin domain-containing protein 3
TLR: Toll-like receptor
TNF-α: Tumor necrosis factor-α.
