# Supplementary material for: Alloantigen-activated (AAA) CD4+ T cells reinvigorate host endogenous T cell immunity to eliminate pre-established tumors in mice
Source: J Exp Clin Cancer Res. 2021 Oct 8;40:314. doi: 10.1186/s13046-021-02102-6 (PMC8499505; doi:10.1186/s13046-021-02102-6)
Supplement: Supplementary file 1 — Additional file 1: Figure S1. Intratumoral injection of activated DCs failed to induce antitumor immunity to eliminate pre-established melanoma. Figure S2. Intratumoral injection of ex vivo activated autologous T cells failed to induce antitumor immunity to eliminate pre-established melanoma. Figure S3. Representative photographs of tumor growths after the AAA-CD4+ T-cell therapy. Figure S4. Intratumoral injection of allogeneic naïve CD4+ T cells and CD3/CD28-Ab-activated allogeneic CD4+ T cells failed to induce antitumor immunity to eliminate pre-established melanoma. Figure S5. Representative scatterplots to detect specific populations of tumor-infiltrating T cells. Figure S6. Increased infiltration and Th1-type inflammation of the host CD4+ T cells after AAA-CD4+ T-cell therapy. Figure S7. Host endogenous CD4+ T cells are required to mediate antitumor immunity in mice undergoing AAA-CD4+ T-cell therapy. Figure S8. Tumor-infiltrating host NK cells are not essential to induce antitumor immunity in AAA-CD4+ T-cell therapy. Figure S9. The number of tumor-infiltrating host B cells after the AAA-CD4+ T-cell therapy. Figure S10. Larger number of host-activated DCs and macrophages were isolated from the draining lymph nodes of the AAA-CD4+group, as compared to auto-CD4+ and control groups at 24 h after therapy. Figure S11. The number of host Tregs in the tumor and peripheral blood after the AAA-CD4+ T-cell therapy. Figure S12. Gene expressions of immunosuppressive biomarkers in the tumor after the AAA-CD4+ T-cell therapy. Figure S13. DCs generated from 129X1 mice that share MHC class II molecules with B6 mice induced AAA-CD4+ T cells elicited antitumor immunity in the B16F1 inoculated B6 mice. Table S1. Primers for real-time RT-PCR. [file 13046_2021_2102_MOESM1_ESM.docx]

**Supplementary materials.**

**Fig. S1. Intratumoral injection of activated DCs failed to induce antitumor immunity to eliminate pre-established melanoma**

B6 mice-derived Flt3L-DCs were activated using LPS and R848 with the lysate of B16F1 cells. Host B6 mice were subcutaneously injected with 1 × 10^6^ B16F1 cells on day zero. 1 x 10^6^ activated Flt3L-DCs were intratumorally injected into the B6 mice nine days after B16F1 inoculation. The PBS-injected group served as the control. Tumor growth (a) and survival (b) of each group are shown.

Abbreviations: N.S., not significant.

**Fig. S2. Intratumoral injection of *ex vivo* activated autologous T cells failed to induce antitumor immunity to eliminate pre-established melanoma**

Host B6 mice were subcutaneously injected with 1 × 10^6^ B16F1 cells on day zero. CD8^+^ and CD4^+^ T cells were isolated from spleens of B6 mice. The CD8^+^ T cells and CD4^+^ T cells were activated in cultures with B6-derived Flt3L-DCs pulsed with human gp100 and lysate of B16F1, respectively. A total of 2 × 10^6^ activated autologous CD8^+^ T cells **(a-b)** and CD4^+^ T cells **(c-d)** were separately injected into the tumors nine days after B16F1 inoculation. The PBS-injected group served as the control. Tumor growth (a, c) and survival (b, d) of each group are shown.

Abbreviations: N.S.: not significant.

**Fig. S3. Representative photographs of tumor growths after the AAA-CD4^+^ T-cell therapy.**

AAA-CD4^+^ T cells were produced from BALB/c mouse CD4^+^ T cells in cultures activated with B6-derived GM-DCs. Host B6 mice were subcutaneously injected with 1 × 10^6^ B16F1 cells on day zero. Nine days after B16F1 inoculation, AAA-CD4^+^ T cells were intratumorally injected (AAA-CD4^+^ group). The PBS-injected group served as the control. Sequential photographs of tumor growth in both groups are shown.

**Fig. S4. Intratumoral injection of allogeneic naïve CD4^+^ T cells and CD3/CD28-Ab-activated allogeneic CD4^+^ T cells failed to induce antitumor immunity to eliminate pre-established melanoma**

Host B6 mice were subcutaneously injected with 1 × 10^6^ B16F1 cells on day zero. CD4^+^ T cells were isolated from spleens of BALB/c mice. **a-b**: 2 × 10^6^ allogeneic naïve CD4^+^ T cells were injected into the tumors nine days after B16F1 inoculation. The PBS-injected group served as the control. Tumor growth (a) and survival (b) are shown. **c-d**: Allogeneic CD4^+^ T cells were activated in cultures containing human IL-2 with mouse CD3 and CD28-Ab for three days. 2 x 10^6^ Ab-activated allogeneic CD4^+^ T cells were injected into the tumors nine days after B16F1 inoculation. The PBS-injected group served as the control. Tumor growth (c) and survival (d) are shown.

Abbreviations: Ab: antibody, N.S.: not significant.

**Fig. S5. Representative scatterplots to detect specific populations of tumor-infiltrating T cells**

Four and twenty-four h after the intratumoral injection of CSFE-labeled *ex vivo* activated CD4^+^ T cells, the mice were euthanized and tumors were resected. Then, mononuclear cells that had infiltrated the tumors were isolated using Percoll density-gradient centrifugation. The isolated cells were stained with fluorescently conjugated antibodies and analyzed using a flow cytometer. Representative scatterplots to detect specific populations of tumor-infiltrating T cells are shown.

**Fig. S6. Increased infiltration and Th1-type inflammation of the host CD4^+^ T cells after AAA-CD4^+^ T-cell therapy**

AAA-CD4^+^ cells were produced from BALB/c mouse CD4^+^ T cells in cultures activated with B6-derived GM-DCs. Host B6 mice were subcutaneously injected with 1 × 10^6^ B16F1 cells on day zero. Nine days after B16F1 inoculation, AAA-CD4^+^ T cells that had been labeled with CFSE were intratumorally injected (AAA-CD4^+^ group). B6-derived DC-activated B6-derived CD4^+^ T cells that had been labeled with CFSE (auto-CD4^+^ group) and PBS-injected mice were used as controls (three mice per group). **a**: Representative scatterplots of the CFSE negative host tumor-infiltrating CD4^+^ T cells are shown. **b**: The number of host CD44^hi^/CD62L^low^ effector memory CD4^+^ T cells in the tumor. **c**: Results show the relative expression of the indicated gene in tumor-infiltrating host CD44^hi^/CD4^+^ T cells, normalized to those in the host CD4^+^ T cells isolated from the spleen of PBS-treated mice. **d**: The number of total host CD4^+^ T cells isolated from DLNs is shown. Data are expressed as mean ± SD. *P <0.05, **P <0.01, ***P <0.001. Representative data from two independent experiments are shown.

Abbreviations: TV: tumor volume.

**Fig. S7. Host endogenous CD4^+^ T cells are required to mediate antitumor immunity in mice undergoing AAA-CD4^+^ T-cell therapy**

AAA-CD4^+^ cells were produced from BALB/c mouse CD4^+^ T cells in cultures activated with B6-derived GM-DCs. Host B6 mice and B6 background CD4 knockout mice were subcutaneously injected with 1 × 10^6^ B16F1 cells on day zero. Nine days after B16F1 inoculation, AAA-CD4^+^ T cells were intratumorally injected into the B6 mice and the CD4 knockout mice. PBS-injected mice were used as controls. Tumor growth (a) and survival (b) are shown. ***P <0.001. The data are pooled from two independent experiments.

**Fig. S8. Tumor-infiltrating host NK cells are not essential to induce antitumor immunity in AAA-CD4^+^ T-cell therapy**

AAA-CD4^+^ T cells were produced from BALB/c mouse CD4^+^ T cells in cultures activated with B6-derived GM-DCs. Host B6 mice were subcutaneously injected with 1 × 10^6^ B16F1 cells on day zero. Nine days after B16F1 inoculation, AAA-CD4^+^ T cells that had been labeled with CFSE were intratumorally injected (AAA-CD4^+^ group). B6-derived DC-activated B6-derived CD4^+^ T cells that had been labeled with CFSE (auto-CD4^+^ group) and PBS-injected mice were used as controls. **a**: The number of host NK1.1^+^ NK cells infiltrating the tumor (three mice per group). Data are expressed as mean ± SD**.** Representative data from two independent experiments are shown. **b-c**: Before injecting the AAA-CD4^+^ T cells, anti-murine NK1.1 eliminating antibody or control IgG was intraperitoneally injected at a dose of 500 μg on day 8 and 250 μg on days 10 and 12. Tumor growth (b) and survival (c) are shown. *P <0.05, **P <0.01, ***P <0.001.

Abbreviations: TV: tumor volume, N.S.: not significant.

**Fig. S9. The number of tumor-infiltrating host B cells after the AAA-CD4^+^ T-cell therapy**

AAA-CD4^+^ T cells were produced from BALB/c mouse CD4^+^ T cells in cultures activated with B6-derived GM-DCs. Host B6 mice were subcutaneously injected with 1 × 10^6^ B16F1 cells on day zero. Nine days after B16F1 inoculation, AAA-CD4^+^ T cells were intratumorally injected (AAA-CD4^+^ group). B6-derived DC-activated B6-derived CD4^+^ T cells (auto-CD4^+^ group) and PBS-injected mice were used as controls (three mice per group). The graph shows the number of host CD19^+^ B cells infiltrating the tumor. Data are expressed as mean ± SD**.** Representative data from two independent experiments are shown.

Abbreviations: TV, tumor volume

**Fig. S10. Larger number of host-activated DCs and macrophages were isolated from the draining lymph nodes of the AAA-CD4^+^ group, as compared to auto-CD4^+^ and control groups at 24 h after therapy**

AAA-CD4^+^ T cells were produced from BALB/c mouse CD4^+^ T cells in cultures activated with B6-derived GM-DCs. Host B6 mice were subcutaneously injected with 1 × 10^6^ B16F1 cells on day zero. Nine days after B16F1 inoculation, AAA-CD4^+^ T cells that had been labeled with CFSE were intratumorally injected into the host B6 mice (AAA-CD4^+^ group). B6-derived DC-activated B6-derived CD4^+^ T cells that had been labeled with CFSE (auto-CD4^+^ group) and PBS-injected mice were used as controls (three mice per group). **a**: The number of host CD11c^+^/MHC-II^hi^/F4/80^-^ DCs isolated from DLNs is shown. **b**: Representative histogram showing the percentage expression of the indicated co-stimulatory molecules on the host CD11c^+^/MHC-II^hi^/F4/80^-^ DCs isolated from DLNs of the AAA-CD4^+^ T-cell group at 24 h after therapy. **c**: The number of host F4/80 positive macrophages that infiltrated into the DLNs is shown. Data are expressed as mean ± SD. *P <0.05, **P <0.01, ***P <0.001. Representative data from two independent experiments are shown.

**Fig. S11. The number of host Tregs in the tumor and peripheral blood after the AAA-CD4^+^ T-cell therapy**

AAA-CD4^+^ T cells were produced from BALB/c mouse CD4^+^ T cells in cultures activated with B6-derived GM-DCs. Host B6 mice were subcutaneously injected with 1 × 10^6^ B16F1 cells on day zero. Nine days after B16F1 inoculation, AAA-CD4^+^ T cells that had been labeled with CFSE were intratumorally injected (AAA-CD4^+^ group). PBS-injected mice were used as controls. 24 h after intratumoral injection of activated CD4^+^ T cells, the mice were euthanized (three mice per group). Immediately after euthanasia, peripheral blood (PB) was collected from the heart, and the tumors were resected. Subsequently, mononuclear cells were isolated from PBs and tumors for flow analysis. The numbers of host TCRβ^+^/CD4^+^/CD25^+^/FoxP3^+^ cells in the PB and in the tumor are shown. Data are expressed as mean ± SD**.** Representative data from two independent experiments are shown. Abbreviations: PB, peripheral blood; TV, tumor volume

**Fig. S12. Gene expressions of immunosuppressive biomarkers in the tumor after the AAA-CD4^+^ T-cell therapy**

AAA-CD4^+^ T cells were produced from BALB/c mouse CD4^+^ T cells in cultures activated with B6-derived GM-DCs. Host B6 mice were subcutaneously injected with 1 × 10^6^ B16F1 cells on day zero. Nine days after B16F1 inoculation, AAA-CD4^+^ T cells were intratumorally injected (AAA-CD4^+^ group). PBS-injected mice were used as controls. 24 h after intratumoral injection of AAA-CD4^+^ T cells, the mice were euthanized, and tumors were resected (three mice per group). Total RNA from the flash-frozen tumor tissues was extracted by homogenization with TRIzol^™^ in liquid nitrogen. Results show relative expression levels of the indicated genes in the whole tumor, normalized to those in vitro cultured B16F1 tumor cells. Data are expressed as mean ± SD**.** **P <0.01, ***P <0.001. Representative data from two independent experiments are shown.

**Fig. S13. DCs generated from 129X1 mice that share MHC class II molecules with B6 mice induced AAA-CD4^+^ T cells elicited antitumor immunity in the B16F1 inoculated B6 mice.**

AAA-CD4^+^ cells were produced from BALB/c mouse CD4^+^ T cells in cultures activated using either B6-derived or 129X1-derived GM-DCs. Host B6 mice were subcutaneously injected with 1 × 10^6^ B16F1 cells on day zero. Nine days after B16F1 inoculation, either AAA-CD4^+^ T cells were intratumorally injected. PBS-injected mice were used as controls. Tumor growth (a) and survival (b) are shown. A mouse in the B6-derived DC activated AAA-CD4^+^ T cell-treated group died due to an unknown cause at day 73 after tumor inoculation. ***P <0.001. The data are pooled from two independent experiments.

| Gene name |  | Primer sequence |
| --- | --- | --- |
| 18S | Forward | 5’- GCTGCTGGCACCAGACTT -3’ |
|  | Reverse | 5’- CGGCTACCACATCCAAGG -3’ |
| GAPDH | Forward | 5’- CGCCGCCATGTTGCA -3’ |
|  | Reverse | 5’- GGAAGGCCTAAGCAAGATTTCA -3’ |
| Ifng | Forward | 5’- AGCTCTTCCTCATGGCTGTT -3' |
|  | Reverse | 5’- TTTGCCAGTTCCTCCAGATA -3' |
| TNFα | Forward | 5’- AAATGGGCTTTCCGAATTCA -3' |
|  | Reverse | 5’- CAGGGAAGAATCTGGAAAGGT -3' |
| IL-2 | Forward | 5’- AACTCCCCAGGATGCTCAC -3' |
|  | Reverse | 5’- CGCAGAGGTCCAAGTTCATC -3' |
| Prf1 | Forward | 5’- GCTCCCACTCCAAGGTAGC -3' |
|  | Reverse | 5’- TTTGTACCAGGCGAAAACTGT -3' |
| Gzmb | Forward | 5’- CCACTCTCGACCCTACATGG -3' |
|  | Reverse | 5’- GGCCCCCAAAGTGACATTTATT -3' |
| IL-1β | Forward | 5’- GCTGAAAGCTCTCCACCTCA -3' |
|  | Reverse | 5’- AGGCCACAGGTATTTTGTCG -3' |
| IL-12p35 | Forward | 5’- CCAGCACATTGAAGACCTGT -3' |
|  | Reverse | 5’- CAGGGTCATCATCAAAGACG -3' |
| IL-23 | Forward | 5’- GACAACAGCCAGTTCTGCT -3' |
|  | Reverse | 5’- AGGGAGGTGTGAAGTTGCTC -3' |
| IL-10 | Forward | 5’- CCCAGAAATCAAGGAGCATT -3' |
|  | Reverse | 5’- TCACTCTTCACCTGCTCCAC -3' |

**Table S1. Primers for real-time RT-PCR**

| Gene name |  | Primer sequence |
| --- | --- | --- |
| 18S | Forward | 5’- GCTGCTGGCACCAGACTT -3’ |
|  | Reverse | 5’- CGGCTACCACATCCAAGG -3’ |
| GAPDH | Forward | 5’- CGCCGCCATGTTGCA -3’ |
|  | Reverse | 5’- GGAAGGCCTAAGCAAGATTTCA -3’ |
| Ifng | Forward | 5’- AGCTCTTCCTCATGGCTGTT -3' |
|  | Reverse | 5’- TTTGCCAGTTCCTCCAGATA -3' |
| TNFα | Forward | 5’- AAATGGGCTTTCCGAATTCA -3' |
|  | Reverse | 5’- CAGGGAAGAATCTGGAAAGGT -3' |
| IL-2 | Forward | 5’- AACTCCCCAGGATGCTCAC -3' |
|  | Reverse | 5’- CGCAGAGGTCCAAGTTCATC -3' |
| Prf1 | Forward | 5’- GCTCCCACTCCAAGGTAGC -3' |
|  | Reverse | 5’- TTTGTACCAGGCGAAAACTGT -3' |
| Gzmb | Forward | 5’- CCACTCTCGACCCTACATGG -3' |
|  | Reverse | 5’- GGCCCCCAAAGTGACATTTATT -3' |
| IL-1β | Forward | 5’- GCTGAAAGCTCTCCACCTCA -3' |
|  | Reverse | 5’- AGGCCACAGGTATTTTGTCG -3' |
| IL-12p35 | Forward | 5’- CCAGCACATTGAAGACCTGT -3' |
|  | Reverse | 5’- CAGGGTCATCATCAAAGACG -3' |
| IL-23 | Forward | 5’- GACAACAGCCAGTTCTGCT -3' |
|  | Reverse | 5’- AGGGAGGTGTGAAGTTGCTC -3' |
| IL-10 | Forward | 5’- CCCAGAAATCAAGGAGCATT -3' |
|  | Reverse | 5’- TCACTCTTCACCTGCTCCAC -3' |

| Gene name |  | Primer sequence |
| --- | --- | --- |
| Gene name |  | Primer sequence |
| ARG1 | Forward | 5’- CAAGACAGGGCTCCTTTCAG -3’ |
|  | Reverse | 5’- AAGCAAGCCAAGGTTAAAGC-3’ |
| iNOS | Forward | 5’- ACCTTGGTGAAGGGACTGAG -3’ |
|  | Reverse | 5’- TCCGTTCTCTTGCAGTTGAC -3’ |
| IDO | Forward | 5’- CAAGACCTGAAAGCATTGGA -3' |
|  | Reverse | 5’- CACAAAGTCACGCATCCTCT -3' |
| COX2 | Forward | 5’- AACCGCATTGCCTCTGAAT-3' |
|  | Reverse | 5’- CATGTTCCAGGAGGATGGAG -3' |
